# Hospital and Institutional News

**Published:** 1920-12-18

**Authors:** 


### HOSPITAL AND INSTITUTIONAL NEWS.

### LORDS AND COMMONS AND THE BILL.

The Ministry of Health (Miscellaneous Provisions) Bill saw the last of the House of Commons for the present about breakfast-time one morning last week, the jaded members having sat up all night to discuss the last clauses during the report stage. Eight of the fourteen clauses that the Government offered to drop remained after all. The chief discussion on the Third Reading had to do with Clause 8, which enables ex-soldiers suffering from shell-shock and other cases of incipient mental disorders of recent origin to be treated in voluntary institutions by private doctors. The clause, amended by the Government to provide that unfit persons who desire to leave such institutions may be detained for a period of forty-eight hours, was carried. Clause 9, which enables county councils to take over Poor-law infirmaries and maintain them as general hospitals, was debated at length and carried also, as was the provision regulating the testing and sale of clinical thermometers. It was announced that 30,000 of these thermometers are tested weekly at Kew at a charge of 3d. each, which will cover the expenditure incurred under the clause. On Tuesday last the Bill came before the House of Lords, sponsored by Lord Sandhurst, and was rejected. In what form legislation will next appear remains to be seen.

### THE MINISTRY OF HEALTH AND VOLUNTARY MATERNITY AND CHILD-WELFARE CENTRES.

Dr. Addison has addressed a circular letter to public authorities throughout the country stating that he has had under consideration the financial position of the centres and other institutions for maternity and child-welfare maintained by voluntary agencies in England. As the local authorities are aware, these institutions are aided by the Ministry with a grant of fifty per cent. of their approved net expenditure, provided they are satisfactorily conducted, and that co-ordination is, as far as practicable, effected with the maternity and child-welfare scheme of the local authorities in whose district they work. The Ministry regrets to learn, however, that owing to the diminution of voluntary subscriptions, the general rise of prices and the development of their activities, many voluntary societies engaged in maternity and child-welfare are in serious financial difficulties, which may in some cases eventually necessitate their closure. The Minister attaches great importance to the work carried on by voluntary societies for maternity and child-welfare, and desires to see it preserved where it is well administered and forms a definite part of the general maternity and child-welfare work of the district, and he is very anxious that all possible steps shall be taken to ensure the continuance of this work. Many of these societies are carrying on institutions which, if they are allowed to lapse, will have to be replaced by institutions wholly financed by the local authority, if the maternity and child-welfare services of the district are to be maintained. The Ministry therefore urges all local authorities carrying out schemes for maternity and child-welfare to consider the question of rendering such financial assistance to the voluntary agencies engaged in the work in their district as the circumstances render desirable. Many local authorities are already doing this on the principle of contributing either a fixed percentage of the expenditure of the voluntary agency or a fixed sum per annum. Any proposal to aid voluntary agencies in this way should first be submitted to the Ministry for sanction, and grants will be payable under the regulations in respect of approved expenditure for the purpose.

### A CONFERENCE OF HOSPITAL STAFFS.

In view of the importance to the medical profession itself of the situation arising from Dr. Addison's agreement to appoint "a small committee to inquire into the financial position of the voluntary hospitals throughout the country and to make recommendations," the British Medical Association has resolved to call a conference of representatives of the staffs of voluntary hospitals at 11 a.m. on Tuesday, December 21, in London. The medical secretary has written to the honorary secretaries of the staffs of most of the larger hospitals inviting each to nominate a physician and a surgeon to represent it at the conference. According to the *British Medical Journal* the questions to be discussed include: (1) What is the general opinion of members of hospital staffs as to how the present financial difficulties should be met?—and possibly to make arrangements for witnesses to give evidence before the Government Committee. (2) What should be the attitude of members of hospital staffs in the event of decisions being taken which would lead to patients paying, in part or in whole, the hospital maintenance fees, either individually, or by means of some contributory method, or with the addition of rate aid or State aid, or by a combination of two or more of these methods? In particular it should be considered in what circumstances it would be necessary to insist upon payment being made to the members of the staffs for professional services.

### THE FUTURE OF THE POOR-LAW INFIRMARY.

As we go to press there is being held at the meeting of the Harveian Society, 11 Chandos Street, a discussion upon what the future has in store for the Poor-law infirmaries and the conditions and circumstances in which the greatest national benefit can be derived from these institutions. Drs. C. M. Wilson and Charles Buttar and Mr. E. W. Morris, the well-known house-governor of the London Hospital, are, we understand, among those who will lead the discussion. Some valuable opinions should be forthcoming, and we hope to deal with some aspects of this important problem in our next issue.

B

### KING EDWARD'S HOSPITAL FUND FOR LONDON.

On page 265 will be found a report of the meeting of the General Council of King Edward's Hospital Fund when the usual Christmas grants were announced. The total grants on this occasion for maintenance and capital charges amount to £190,000, or £40,000 less than last year, but it must not be forgotten that an emergency grant of £450,000 was made earlier in the year, as well as the distribution of £250,000 of Red Cross money given for building, etc. A return of the exact figures of expenditure and income up to September 30 was before the Fund when the distribution list was drawn up, so we can be sure that the immediate needs of the institutions were considered in each case. We hope that the Fund will continue to avail themselves in the future of recent figures, instead of, as in past years, relying upon a financial statement nearly twelve months old.

### BRITISH HOSPITALS ASSOCIATION.

A correspondent in our last issue called attention to the fact that the constituent hospitals of London who send representatives to the London Regional Committee of the British Hospitals Association had been notified that these members should be of the governing body. We understand that this qualification, which was not covered by the resolution of the members at the general meeting on December 2, has been withdrawn, and the hospitals have been informed that they may send as representatives both members of committee and executive officers. We hope there will be substantial representation of both classes.

### TRAGIC DEATH OF A SECRETARY.

We much regret to report the death of Mr. Guy B. Dale, the secretary of the Metropolitan Hospital, which occurred on Monday evening, being the result of a compound fracture of the leg, followed by gangrene. Mr. Dale was involved in a motor accident on Saturday last on the way to the Cranbrook Convalescent Home of the Metropolitan Hospital. He was one of the youngest secretaries in London, being only twenty-six years of age, and he last year succeeded Mr. J. Courtney Buchanan, who was then appointed secretary to the Cancer Hospital. Mr. Dale was very well known in the football field, being one of the most brilliant players we possess, and his career as a hospital secretary had every sign of becoming equally brilliant. Mr. Dale was formerly secretary of the Beckett Hospital, Barnsley.

### A SUCCESSFUL MATINEE.

The *matinée* recently held in aid of the Royal Medical Benevolent Fund Guild at His Majesty's Theatre was, both artistically and financially, a great success. H.M. the Queen of Spain and H.R.H. Princess Christian graced the Royal Box, and received a warm welcome from a crowded house. The excellent programme arranged was carried through with commendable punctuality, and afforded an example of just what a charity *matinée* performance should be. Miss Winifred Emery made a most welcome return to the stage with Mr. Gerald du Maurier in a one-act play entitled "The Stepmother," Miss Fay Compton and Mr. Basil Rathbone gave a charming rendering of the balcony scene from "Romeo and Juliet," and Mr. Leon M. Lion's company contributed the second act of "The Right to Strike," a play which excited so much interest among the public in general and the medical profession in particular. We are glad to be able to state that the Guild is already richer by well over £1,000, and that, in addition, it is to be congratulated upon the wider publicity which the *matinée* has given to the needs of this branch of our own professional charity.

### SPLENDID RESPONSE TO AN APPEAL FROM GUY'S.

The Governors of Guy's Hospital are to be congratulated on the initial success that has resulted from their appeal to the leading business firms in the boroughs adjacent to the hospital for additional financial help. Before making a wider appeal the Governors felt that those in the immediate neighbourhood of the hospital should first be asked to give their aid, and it is gratifying to find that as a result £1,455 5s. has been sent in as subscriptions, and £7,300 2s. 7d. as donations, or a total of £8,755 7s. 7d. The Prince of Wales heads the subscription list with a gift of twenty guineas, and he has expressed his entire approval of the preliminary lists, which have been submitted to him. The existing annual subscription list of £3,500 is totally inadequate to the immense work carried on at Guy's, with its 613 beds, in which 8,702 patients were treated in 1919, in addition to 437,916 out-patients' attendances, and 62,288 casualty cases. When it is borne in mind that the annual expenditure has increased from £76,000 in 1914 to £128,000 in 1919, with a further increase in 1920, it will readily be seen that Guy's, like the rest of the great London hospitals, must have more assistance from the public. It is to be hoped that this preliminary list of new subscribers and donors will be increased by those who so far have not responded to the appeal. Whilst these amounts come exclusively from employers it is to be hoped that their employees, who benefit from the hospital, will be induced to give their mites towards the hospital funds.

### COUNTRY HOSPITALS "PAY THEIR WAY."

Mr. George Turner, chairman of the general committee of Addenbrooke's Hospital, Cambridge, has written to the *Daily Telegraph* a letter to show Londoners that "even under present conditions it is possible for county hospitals to pay their way." Addenbrooke's, which has 194 beds, spent last year £25,000, and suffered, says Mr. Turner, a deficit of "less than £500, while by earmarked donations and endowment legacies we have increased our invested capital." This very satisfactory result Mr. Turner attributes to "increased efforts on the part of the working classes," chiefly in the direction of small weekly payments and Friendly Societies' Sunday parades. The hospital is unable by its present rules to make a charge on patients toward the cost of maintenance, but "a substantial sum has been realised by voluntary thankofferings from patients." We believe that these two results are naturally connected. The hospital presided over the formation of the Cambridge and District Workers' Hospital Fund, and did its best to encourage the movement, which was one of the early attempts to secure weekly contributions in a county town. Mr. R. J. Coles, now secretary of King's College Hospital, London, worked very hard at Addenbrooke's to secure the success of the fund, and we believe that Addenbrooke's is now reaping the reward of its previous initiative, for, as we have already said, it was a new departure to transfer the workers' movement from an industrial city to a county and university town. If more chairmen of country hospitals would enlighten Londoners of the position of their hospitals, there would be less ignorance than there is in the Metropolis of the vitality of the voluntary system. We hope that Mr. Turner's excellent lead will be followed elsewhere.

### THE VALUE OF ADVERTISEMENT.

In connection with a recent article upon the relative value of advertisement and of publicity to hospitals, we notice that the *Daily Chronicle* declares that the success of the workers' contributions to the Leicester Royal Infirmary, which now amount to over £20,000 a year, dates from "a scheme to advertise the hospital" in 1903. Our contemporary alludes to the appointment of a full-time organiser and secretary to visit factories, so as to induce the workers to contribute 1d. a week. This is a good instance of the confusion between advertisement and publicity. We should like to hear from Mr. Harry Johnson, the house governor, whether he agrees that the term "advertisement" is correctly applied to the methods by which the success of the workers' fund at Leicester has been brought about.

### HALIFAX HELPING ITSELF.

When Halifax is in difficulties it usually manages in some way or other to extricate itself. In order to make up a deficit on the last year's working expenses the Royal Infirmary set about arranging a three days' bazaar, to be held in the Victoria Hall, to raise £3,000. This figure seemed rather ambitious, but on Thursday of last week the treasurer received no less than £6,970! But this is not all; incidentally, through the bazaar the Halifax Football Club handed over £773, and from private subscriptions came the sum of £1,400!

### THE PRESIDENT OF CUMBERLAND INFIRMARY.

Dr. Barnes has been elected President of the Cumberland Infirmary. It is an interesting election because not only has he been a member of the staff for forty-seven years, but his family is associated with the hospital. Its first physician was another Dr. Barnes, the uncle of the new President, and it was largely due to his uncle's interest that Dr. Barnes in his student days was led to look forward to becoming a member of the staff. Acknowledging his election, at the recent meeting, Dr. Barnes outlined the financial anxieties of the institution, which had culminated in a present debt of £10,000. But with the difficulties Dr. Barnes has seen the infirmary continue to grow, and we have little doubt that it will maintain this record under his presidency. He has already been Chairman of the Committee of Management, and acted as Secretary to the Extension Committee when the latest enlargement was carried out.

### A MODEL PIECE OF PUBLICITY.

An interesting article upon Moorfields Eye Hospital appeared in the *Daily Telegraph* of December 4. No better illustration could be found of publicity in contrast to advertisement: a subject whereon an article appeared in The Hospital last week. The article, which was headed "Eye Surgery: A Great Ophthalmic Hospital," began by tracing contagious ophthalmia to the troops under Sir Ralph Abercrombie, who followed in Napoleon's footsteps and contracted the disease in the East. In 1802, we are reminded, eye disease was common in England and little was known of it except by quacks. Then the young surgeon, John Cunningham Saunders, anxious to study the disease, founded Moorfields Eye Hospital, which began to receive patients in 1805. Its fame has survived a change of site from Moorfields to the City Road, where the Royal London Ophthalmic Hospital continues to be known by its original title. From the parent institution its offshoots in America, Canada, Egypt, Ceylon, and Australia, among others, are mentioned. The operation for cataract was first performed at Moorfields Eye Hospital. A good and quiet description of the in- and out-patient departments then follows, with a word on Helmholtz. The financial position is incidentally summarised, and the article ends with the news that two benefactors have given £5,000 to enable the work to be carried on, and that £100,000 is now being asked for. The article is a model piece of work, and we believe that the London dailies would welcome articles of the same kind from other institutions if the right pen could be found to write them. The article itself should be studied as an example of journalistic publicity, and many hints can be gained from it in the arts of presentation and of interesting readers in hospital work.

### A PRESENTATION.

Mr. W. H. Harper, House Governor and Secretary of the Wolverhampton and Staffordshire General Hospital, completed twenty-one years' service on December 1. At a meeting of the Board of Management held on Tuesday, November 30, the occasion was marked by a presentation to Mr. Harper by the chairman and members of a silver fruit- or flower-bowl and a pair of silver flower-vases. The bowl was suitably inscribed, and referred to the loyal and efficient services rendered to the hospital by the recipient. A further presentation was made to Mr. Harper on Saturday evening last at a smoking concert held by the Hospital Saturday

Committee of the hospital. The present from this Committee took the form of a handsome smoking-cabinet. Mr. Harper is well known in hospital circles. He is a member of the British Hospitals Association, honorary secretary to the newly-formed Midland Regional Committee of that Association, and is this year President of the Midlands Branch of the Hospital Officers' Association.

### ROYAL HANTS COUNTY HOSPITAL.

The Treasurer, Mr. L. B. Keyser, was able to give a more satisfactory account of the finances of the Royal Hants County Hospital at the recent Court of Governors, but said that although the revenue is improving the income has not yet overtaken the expenditure. However, he is most hopeful of the future. Mr. Keyser deplored Lord Knutsford's S.O.S. to the Government, but agreed that the Chairman of the London Hospital is right if he means that if hospitals continue on the old lines of depending for income upon one class and one class alone of subscribers they will soon be bankrupt. It is necessary to break fresh ground and to look for support from other sources, such as payments by patients and weekly contributions from workmen. It is to be understood that the workmen who thus contribute are ensuring themselves for free hospital treatment. Employers and insurance companies hardly realise the importance of the work hospitals are doing for them. It must be to the employers' interest that a sick man should be restored to health quickly, and insurance companies must be saving a huge sum annually by the lives which hospitals save and by the skill of the surgeon and physicians in preventing injuries becoming permanent disablement.

### THE EMPLOYEES' FUND AT LEEDS.

Mr. T. F. Braime's scheme for an employees' contribution fund to the Leeds General Infirmary has received the support of 350 firms, and the infirmary will receive about £4,500 per annum thereby. But the annual deficit is £25,000, and to meet it Mr. Braime has two proposals. The first is to induce the remaining firms to subscribe to the employees' contribution fund. Did all these firms in the city and district join the scheme, it would produce £15,000 a year. To get the remaining £10,000 the city is to be divided into areas, with two gentlemen in charge of each, and all the shops, warehouses, and offices in each area will be approached for regular contributions. There can be no doubt that the success of this second plan depends upon the completion of the first. So far about one-third of all the firms in the city and district have joined; it is the remaining two-thirds which have to be secured. Mr. Braime's scheme has done much, not only for the Leeds General Infirmary, but by inducing other centres to follow the plan drafted by him—a plan so well thought out in all its aspects that we gave it in full in The Hospital early in the present year. We watch its progress with the greatest interest, and trust that Mr. Braime will complete the success which he began.

### BECKETT HOSPITAL AND DISPENSARY, BARNSLEY.

The Beckett Hospital and Dispensary, Barnsley, are to be congratulated upon the success of His Worship the Mayor of Barnsley, Col. W. E. Raley's appeal for £20,000. The origin of the appeal was to liquidate the debt of £16,000 incurred chiefly during the war years. Efforts were first commenced in the early part of this year, and within the short space of six months the total sum subscribed was £21,306 5s. 5d. The list of contributors is a long and representative one, showing that a generous response was received from all classes. Mr. A. Whitham, the chairman, and Mr. H. F. Rutherford, the secretary, both played a very active part in the organisation of this appeal, and their valuable assistance was referred to by the Mayor when handing this sum over to the treasurer of the hospital. This occasion was made a public function, and the opportunity was taken to dedicate three beds, two endowed by the late "William Carnelley," and one by the late "John Traviss."

### A PATIENT'S GRATITUDE.

Instances are rare where a patient publicly expresses gratitude to a medical man for professional kindness and devotion to him, it is pleasing therefore to read in a local paper's memoir to the late Dr. Golding Bird Collet the expressions of a former patient, who tells in the form of a letter to the editor how Dr. Collet, on being called to see him when the patient was a younger man and living alone in lodgings, and finding him delirious with an acute disease, had him removed to his own house, placed in a room next his own, and himself nursed him back to health. Dr. Collet was for many years on the staff of the Worthing Hospital.

### NEW HOSPITAL ON MARLOW HILL.

On a site at Marlow Hill, given by Lord Lincolnshire, a new hospital is to be built, to replace the Earl of Beaconsfield Memorial Cottage Hospital at High Wycombe. The money in hand amounts to £31,200, and it is now hoped to secure the rest of the £40,000 required. The new hospital will be the war memorial of the town and district, from which general support should be forthcoming, since it is now largely industrial.

### THIS WEEK'S DRUG MARKET.

There is no change in the state of business either for better or for worse, and there is nothing to indicate that an improvement is likely in the immediate future. Practically all the price alterations are in a downward direction. Castor oil is again cheaper. Nearly all the synthetic drugs are inclined towards lower prices, and with supplies increasing and the demand almost at a standstill it is difficult to see how this condition can be altered for the present. Citric acid is again lower, and the demand is very quiet; the same applies to tartaric acid. There is a good supply of vegetable drugs, and of some a large accumulation; ipecacuanha, squill, sarsaparilla, orris root, and several others are rather cheaper in face of a quiet demand. Camphor, menthol and japanese peppermint oil are very quiet.

